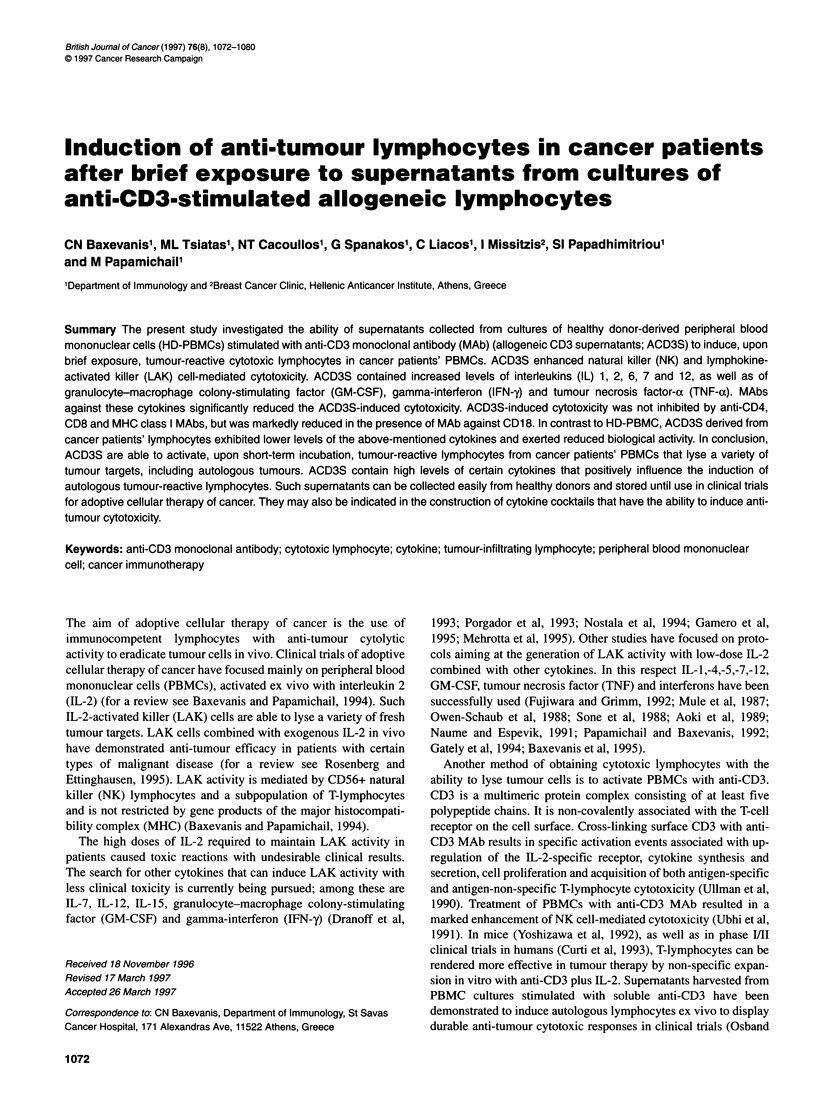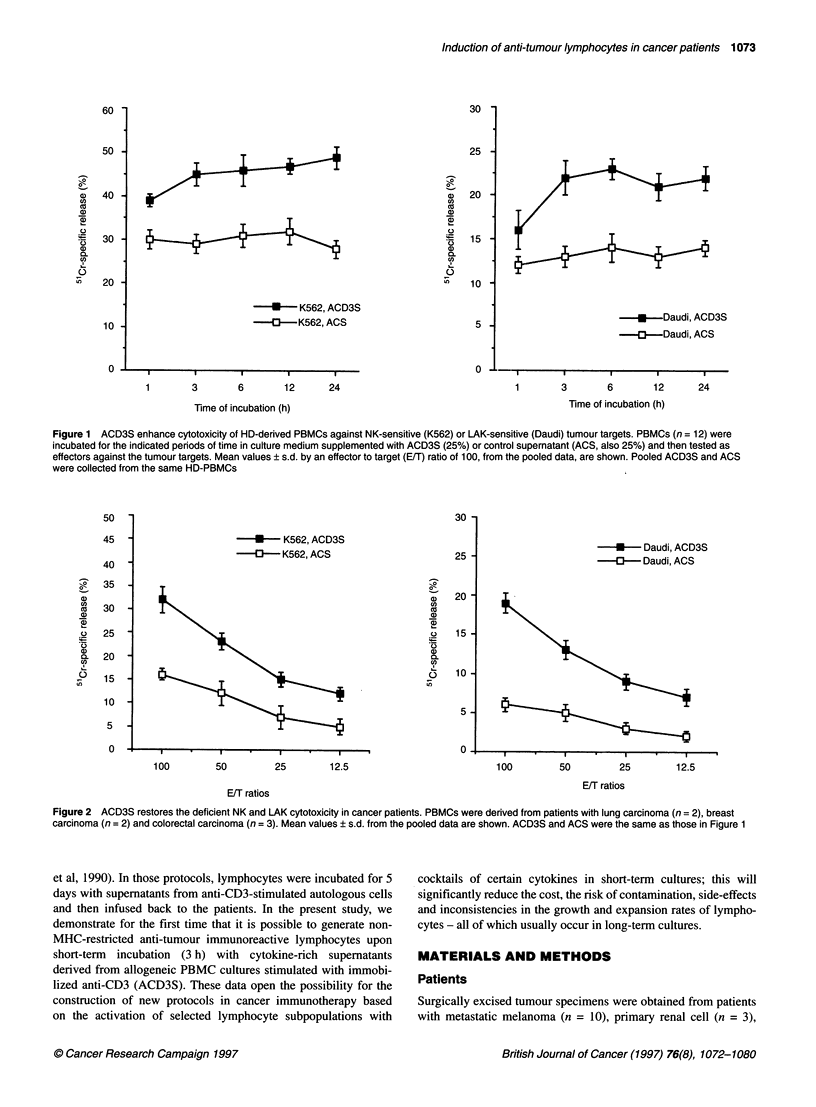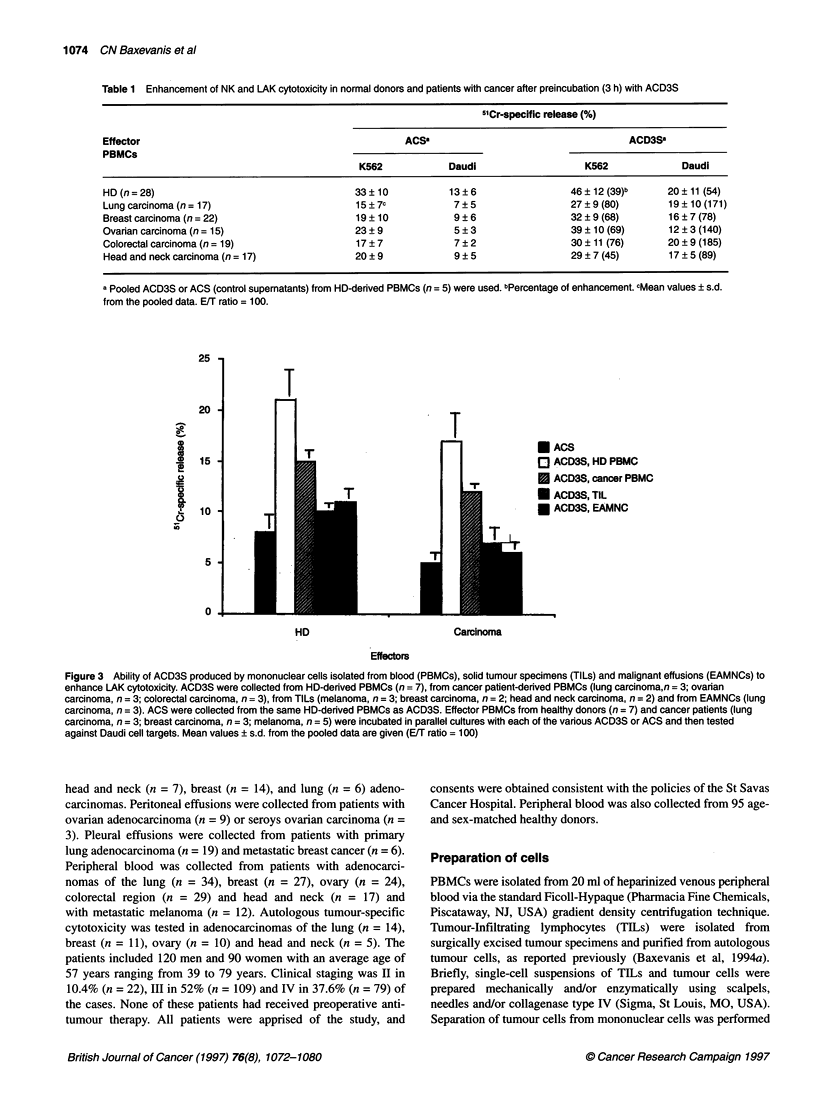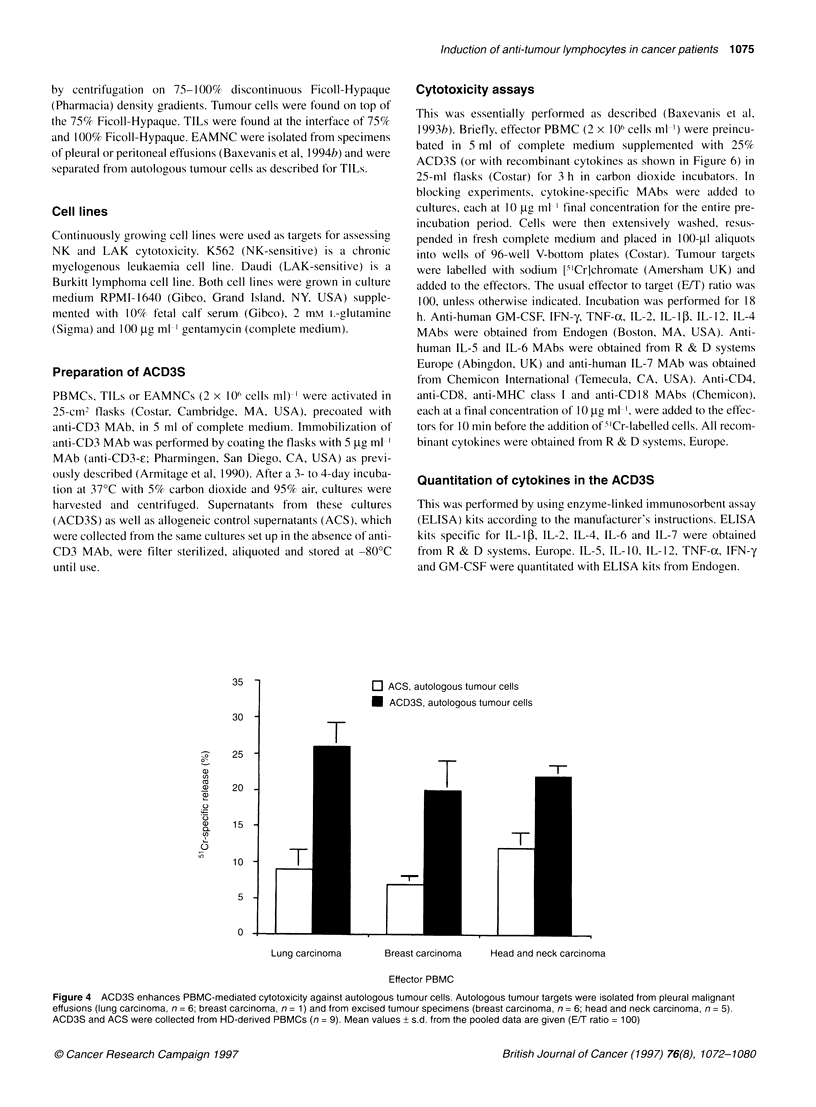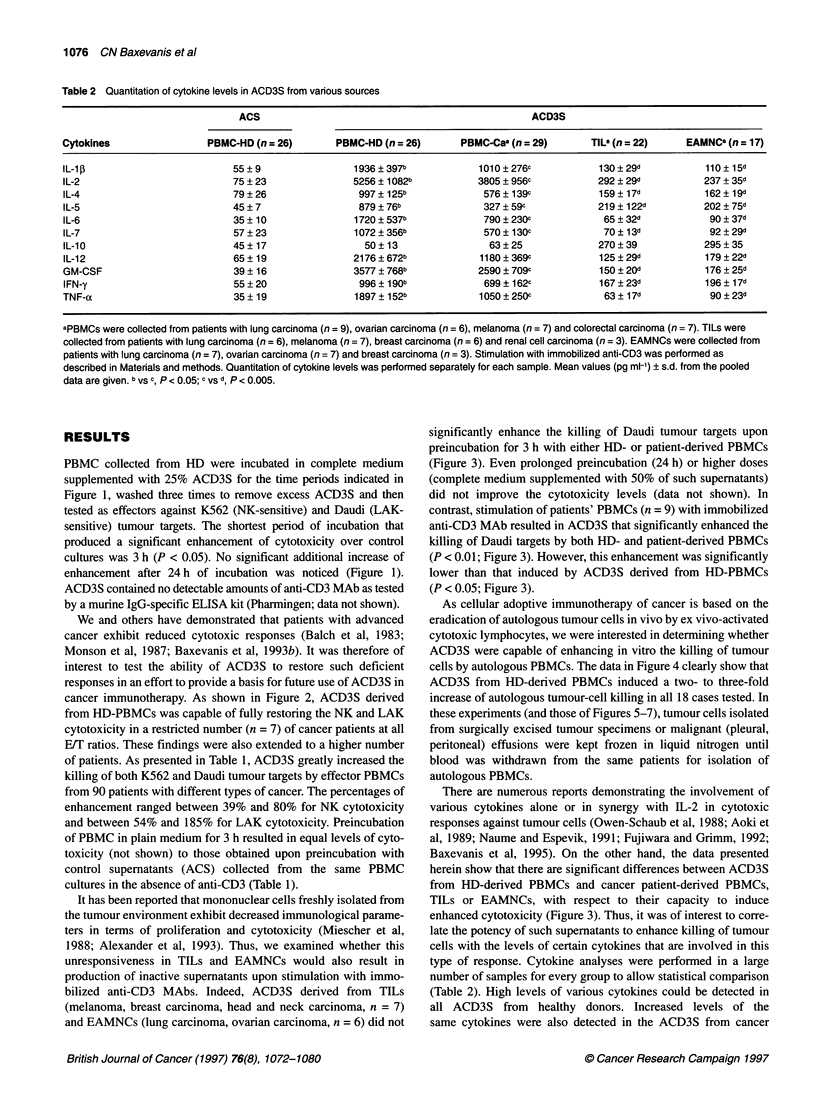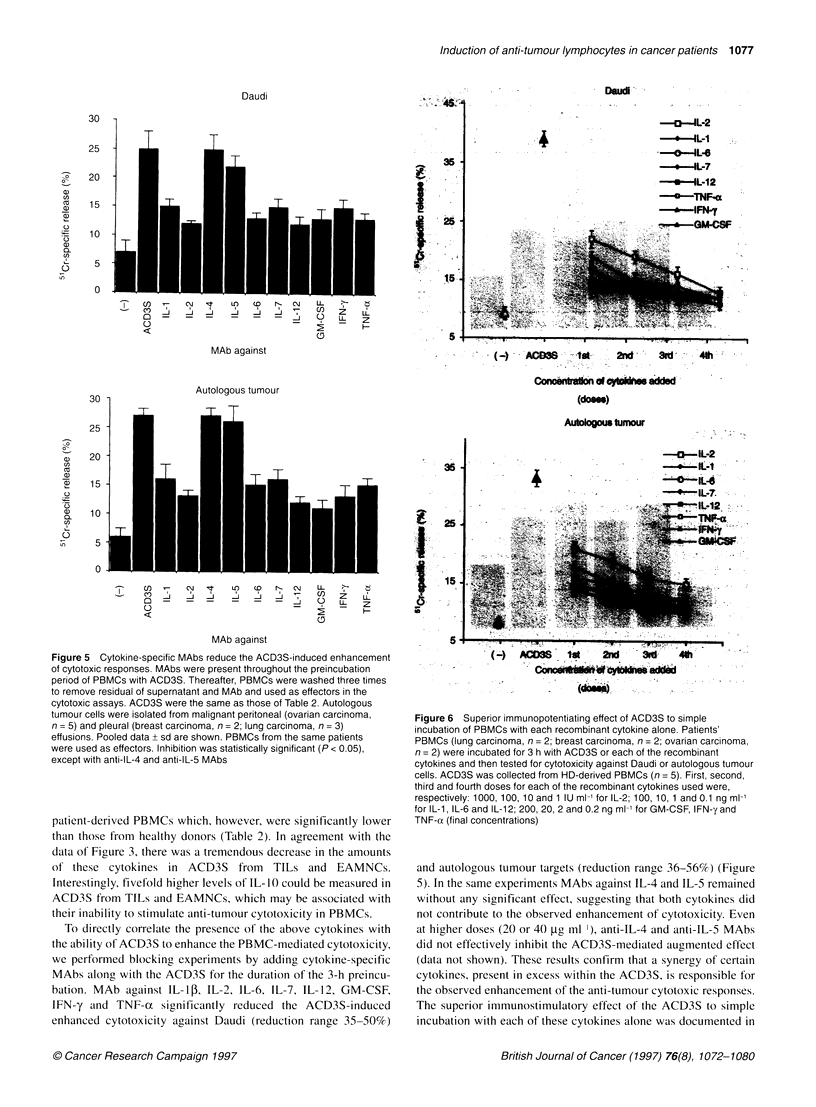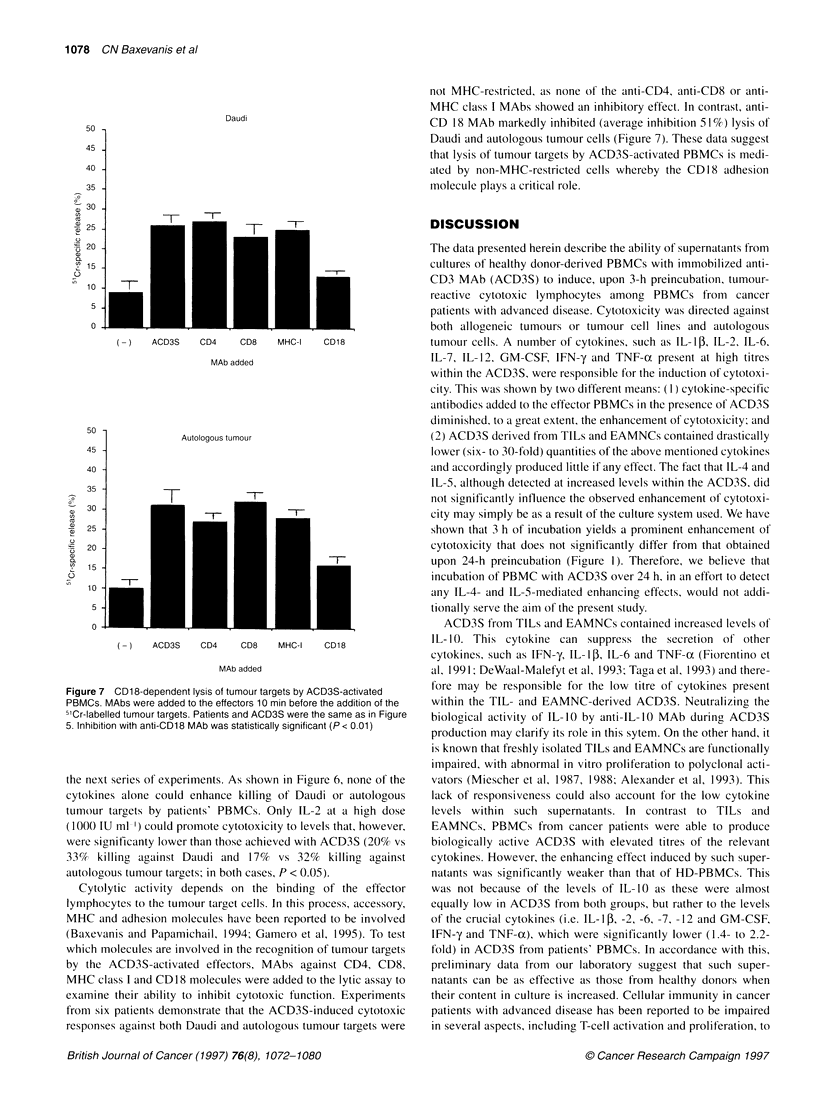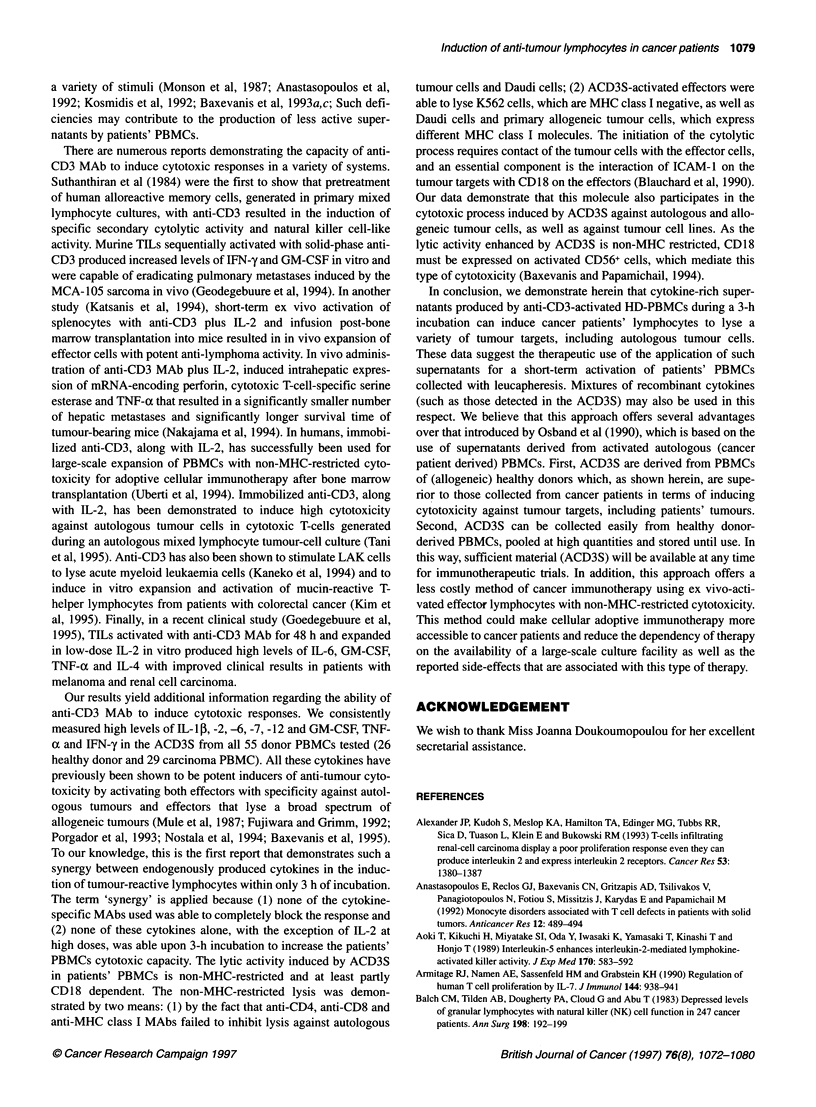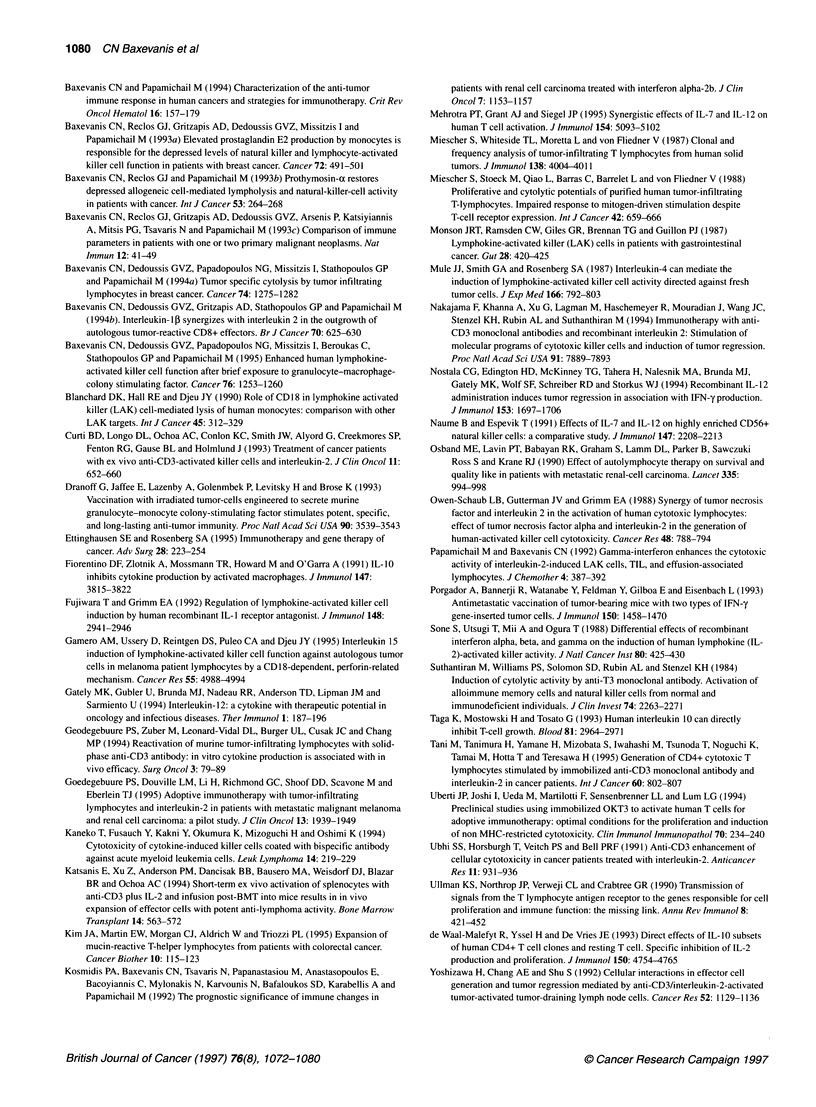# Induction of anti-tumour lymphocytes in cancer patients after brief exposure to supernatants from cultures of anti-CD3-stimulated allogeneic lymphocytes.

**DOI:** 10.1038/bjc.1997.510

**Published:** 1997

**Authors:** C. N. Baxevanis, M. L. Tsiatas, N. T. Cacoullos, G. Spanakos, C. Liacos, I. Missitzis, S. I. Papadhimitriou, M. Papamichail

**Affiliations:** Department of Immunology, Hellenic Anticancer Institute, Athens, Greece.

## Abstract

The present study investigated the ability of supernatants collected from cultures of healthy donor-derived peripheral blood mononuclear cells (HD-PBMCs) stimulated with anti-CD3 monoclonal antibody (MAb) (allogeneic CD3 supernatants; ACD3S) to induce, upon brief exposure, tumour-reactive cytotoxic lymphocytes in cancer patients' PBMCs. ACD3S enhanced natural killer (NK) and lymphokine-activated killer (LAK) cell-mediated cytotoxicity. ACD3S contained increased levels of interleukins (IL) 1, 2, 6, 7 and 12, as well as of granulocyte-macrophage colony-stimulating factor (GM-CSF), gamma-interferon (IFN-gamma) and tumour necrosis factor-alpha (TNF-alpha). MAbs against these cytokines significantly reduced the ACD3S-induced cytotoxicity. ACD3S-induced cytotoxicity was not inhibited by anti-CD4, CD8 and MHC class I MAbs, but was markedly reduced in the presence of MAb against CD18. In contrast to HD-PBMC, ACD3S derived from cancer patients' lymphocytes exhibited lower levels of the above-mentioned cytokines and exerted reduced biological activity. In conclusion, ACD3S are able to activate, upon short-term incubation, tumour-reactive lymphocytes from cancer patients' PBMCs that lyse a variety of tumour targets, including autologous tumours. ACD3S contain high levels of certain cytokines that positively influence the induction of autologous tumour-reactive lymphocytes. Such supernatants can be collected easily from healthy donors and stored until use in clinical trials for adoptive cellular therapy of cancer. They may also be indicated in the construction of cytokine cocktails that have the ability to induce anti-tumour cytotoxicity.


British Journal of Cancer (1997) 76(8), 1072–1080
© 1997 Cancer Research Campaign

### Induction of anti-tumour lymphocytes in cancer patients after brief exposure to supernatants from cultures of anti-CD3-stimulated allogeneic lymphocytes

**CN Baxevanis[1], ML Tsiatas[1], NT Cacoullos[1], G Spanakos[1], C Liacos[1], I Missitzis[2], SI Papadhimitriou[1] and M Papamichail[1]**

[1]Department of Immunology and [2]Breast Cancer Clinic, Hellenic Anticancer Institute, Athens, Greece

**Summary** The present study investigated the ability of supernatants collected from cultures of healthy donor-derived peripheral blood mononuclear cells (HD-PBMCs) stimulated with anti-CD3 monoclonal antibody (MAb) (allogeneic CD3 supernatants; ACD3S) to induce, upon brief exposure, tumour-reactive cytotoxic lymphocytes in cancer patients' PBMCs. ACD3S enhanced natural killer (NK) and lymphokine-activated killer (LAK) cell-mediated cytotoxicity. ACD3S contained increased levels of interleukins (IL) 1, 2, 6, 7 and 12, as well as of granulocyte–macrophage colony-stimulating factor (GM-CSF), gamma-interferon (IFN-γ) and tumour necrosis factor-α (TNF-α). MAbs against these cytokines significantly reduced the ACD3S-induced cytotoxicity. ACD3S-induced cytotoxicity was not inhibited by anti-CD4, CD8 and MHC class I MAbs, but was markedly reduced in the presence of MAb against CD18. In contrast to HD-PBMC, ACD3S derived from cancer patients' lymphocytes exhibited lower levels of the above-mentioned cytokines and exerted reduced biological activity. In conclusion, ACD3S are able to activate, upon short-term incubation, tumour-reactive lymphocytes from cancer patients' PBMCs that lyse a variety of tumour targets, including autologous tumours. ACD3S contain high levels of certain cytokines that positively influence the induction of autologous tumour-reactive lymphocytes. Such supernatants can be collected easily from healthy donors and stored until use in clinical trials for adoptive cellular therapy of cancer. They may also be indicated in the construction of cytokine cocktails that have the ability to induce anti-tumour cytotoxicity.

**Keywords:** anti-CD3 monoclonal antibody; cytotoxic lymphocyte; cytokine; tumour-infiltrating lymphocyte; peripheral blood mononuclear cell; cancer immunotherapy

The aim of adoptive cellular therapy of cancer is the use of immunocompetent lymphocytes with anti-tumour cytolytic activity to eradicate tumour cells in vivo. Clinical trials of adoptive cellular therapy of cancer have focused mainly on peripheral blood mononuclear cells (PBMCs), activated ex vivo with interleukin 2 (IL-2) (for a review see Baxevanis and Papamichail, 1994). Such IL-2-activated killer (LAK) cells are able to lyse a variety of fresh tumour targets. LAK cells combined with exogenous IL-2 in vivo have demonstrated anti-tumour efficacy in patients with certain types of malignant disease (for a review see Rosenberg and Ettinghausen, 1995). LAK activity is mediated by CD56+ natural killer (NK) lymphocytes and a subpopulation of T-lymphocytes and is not restricted by gene products of the major histocompatibility complex (MHC) (Baxevanis and Papamichail, 1994).

The high doses of IL-2 required to maintain LAK activity in patients caused toxic reactions with undesirable clinical results. The search for other cytokines that can induce LAK activity with less clinical toxicity is currently being pursued; among these are IL-7, IL-12, IL-15, granulocyte–macrophage colony-stimulating factor (GM-CSF) and gamma-interferon (IFN-γ) (Dranoff et al, 1993; Porgador et al, 1993; Nostala et al, 1994; Gamero et al, 1995; Mehrotta et al, 1995). Other studies have focused on protocols aiming at the generation of LAK activity with low-dose IL-2 combined with other cytokines. In this respect IL-1,-4,-5,-7,-12, GM-CSF, tumour necrosis factor (TNF) and interferons have been successfully used (Fujiwara and Grimm, 1992; Mule et al, 1987; Owen-Schaub et al, 1988; Sone et al, 1988; Aoki et al, 1989; Naume and Espevik, 1991; Papamichail and Baxevanis, 1992; Gately et al, 1994; Baxevanis et al, 1995).

Another method of obtaining cytotoxic lymphocytes with the ability to lyse tumour cells is to activate PBMCs with anti-CD3. CD3 is a multimeric protein complex consisting of at least five polypeptide chains. It is non-covalently associated with the T-cell receptor on the cell surface. Cross-linking surface CD3 with anti-CD3 MAb results in specific activation events associated with up-regulation of the IL-2-specific receptor, cytokine synthesis and secretion, cell proliferation and acquisition of both antigen-specific and antigen-non-specific T-lymphocyte cytotoxicity (Ullman et al, 1990). Treatment of PBMCs with anti-CD3 MAb resulted in a marked enhancement of NK cell-mediated cytotoxicity (Ubhi et al, 1991). In mice (Yoshizawa et al, 1992), as well as in phase I/II clinical trials in humans (Curti et al, 1993), T-lymphocytes can be rendered more effective in tumour therapy by non-specific expansion in vitro with anti-CD3 plus IL-2. Supernatants harvested from PBMC cultures stimulated with soluble anti-CD3 have been demonstrated to induce autologous lymphocytes ex vivo to display durable anti-tumour cytotoxic responses in clinical trials (Osband

*Received 18 November 1996*
*Revised 17 March 1997*
*Accepted 26 March 1997*

*Correspondence to*: CN Baxevanis, Department of Immunology, St Savas Cancer Hospital, 171 Alexandras Ave, 11522 Athens, Greece

**Figure 1** ACD3S enhance cytotoxicity of HD-derived PBMCs against NK-sensitive (K562) or LAK-sensitive (Daudi) tumour targets. PBMCs ($n$ = 12) were incubated for the indicated periods of time in culture medium supplemented with ACD3S (25%) or control supernatant (ACS, also 25%) and then tested as effectors against the tumour targets. Mean values ± s.d. by an effector to target (E/T) ratio of 100, from the pooled data, are shown. Pooled ACD3S and ACS were collected from the same HD-PBMCs

**Figure 2** ACD3S restores the deficient NK and LAK cytotoxicity in cancer patients. PBMCs were derived from patients with lung carcinoma ($n$ = 2), breast carcinoma ($n$ = 2) and colorectal carcinoma ($n$ = 3). Mean values ± s.d. from the pooled data are shown. ACD3S and ACS were the same as those in Figure 1

et al, 1990). In those protocols, lymphocytes were incubated for 5 days with supernatants from anti-CD3-stimulated autologous cells and then infused back to the patients. In the present study, we demonstrate for the first time that it is possible to generate non-MHC-restricted anti-tumour immunoreactive lymphocytes upon short-term incubation (3 h) with cytokine-rich supernatants derived from allogeneic PBMC cultures stimulated with immobilized anti-CD3 (ACD3S). These data open the possibility for the construction of new protocols in cancer immunotherapy based on the activation of selected lymphocyte subpopulations with cocktails of certain cytokines in short-term cultures; this will significantly reduce the cost, the risk of contamination, side-effects and inconsistencies in the growth and expansion rates of lymphocytes – all of which usually occur in long-term cultures.

### MATERIALS AND METHODS

### Patients

Surgically excised tumour specimens were obtained from patients with metastatic melanoma ($n$ = 10), primary renal cell ($n$ = 3),

© Cancer Research Campaign 1997

**Table 1** Enhancement of NK and LAK cytotoxicity in normal donors and patients with cancer after preincubation (3 h) with ACD3S

| Effector PBMCs | $^{51}$Cr-specific release (%) | | | |
|---|---|---|---|---|
| | ACS[a] | | ACD3S[a] | |
| | K562 | Daudi | K562 | Daudi |
| HD ($n$ = 28) | 33 ± 10 | 13 ± 6 | 46 ± 12 (39)[b] | 20 ± 11 (54) |
| Lung carcinoma ($n$ = 17) | 15 ± 7[c] | 7 ± 5 | 27 ± 9 (80) | 19 ± 10 (171) |
| Breast carcinoma ($n$ = 22) | 19 ± 10 | 9 ± 6 | 32 ± 9 (68) | 16 ± 7 (78) |
| Ovarian carcinoma ($n$ = 15) | 23 ± 9 | 5 ± 3 | 39 ± 10 (69) | 12 ± 3 (140) |
| Colorectal carcinoma ($n$ = 19) | 17 ± 7 | 7 ± 2 | 30 ± 11 (76) | 20 ± 9 (185) |
| Head and neck carcinoma ($n$ = 17) | 20 ± 9 | 9 ± 5 | 29 ± 7 (45) | 17 ± 5 (89) |

[a] Pooled ACD3S or ACS (control supernatants) from HD-derived PBMCs ($n$ = 5) were used. [b]Percentage of enhancement. [c]Mean values ± s.d. from the pooled data. E/T ratio = 100.

**Figure 3** Ability of ACD3S produced by mononuclear cells isolated from blood (PBMCs), solid tumour specimens (TILs) and malignant effusions (EAMNCs) to enhance LAK cytotoxicity. ACD3S were collected from HD-derived PBMCs ($n$ = 7), from cancer patient-derived PBMCs (lung carcinoma,$n$ = 3; ovarian carcinoma, $n$ = 3; colorectal carcinoma, $n$ = 3), from TILs (melanoma, $n$ = 3; breast carcinoma, $n$ = 2; head and neck carcinoma, $n$ = 2) and from EAMNCs (lung carcinoma, $n$ = 3). ACS were collected from the same HD-derived PBMCs as ACD3S. Effector PBMCs from healthy donors ($n$ = 7) and cancer patients (lung carcinoma, $n$ = 3; breast carcinoma, $n$ = 3; melanoma, $n$ = 5) were incubated in parallel cultures with each of the various ACD3S or ACS and then tested against Daudi cell targets. Mean values ± s.d. from the pooled data are given (E/T ratio = 100)

head and neck ($n$ = 7), breast ($n$ = 14), and lung ($n$ = 6) adenocarcinomas. Peritoneal effusions were collected from patients with ovarian adenocarcinoma ($n$ = 9) or seroys ovarian carcinoma ($n$ = 3). Pleural effusions were collected from patients with primary lung adenocarcinoma ($n$ = 19) and metastatic breast cancer ($n$ = 6). Peripheral blood was collected from patients with adenocarcinomas of the lung ($n$ = 34), breast ($n$ = 27), ovary ($n$ = 24), colorectal region ($n$ = 29) and head and neck ($n$ = 17) and with metastatic melanoma ($n$ = 12). Autologous tumour-specific cytotoxicity was tested in adenocarcinomas of the lung ($n$ = 14), breast ($n$ = 11), ovary ($n$ = 10) and head and neck ($n$ = 5). The patients included 120 men and 90 women with an average age of 57 years ranging from 39 to 79 years. Clinical staging was II in 10.4% ($n$ = 22), III in 52% ($n$ = 109) and IV in 37.6% ($n$ = 79) of the cases. None of these patients had received preoperative antitumour therapy. All patients were apprised of the study, and consents were obtained consistent with the policies of the St Savas Cancer Hospital. Peripheral blood was also collected from 95 age- and sex-matched healthy donors.

### Preparation of cells

PBMCs were isolated from 20 ml of heparinized venous peripheral blood via the standard Ficoll-Hypaque (Pharmacia Fine Chemicals, Piscataway, NJ, USA) gradient density centrifugation technique. Tumour-Infiltrating lymphocytes (TILs) were isolated from surgically excised tumour specimens and purified from autologous tumour cells, as reported previously (Baxevanis et al, 1994*a*). Briefly, single-cell suspensions of TILs and tumour cells were prepared mechanically and/or enzymatically using scalpels, needles and/or collagenase type IV (Sigma, St Louis, MO, USA). Separation of tumour cells from mononuclear cells was performed

 © Cancer Research Campaign 1997

by centrifugation on 75–100% discontinuous Ficoll-Hypaque (Pharmacia) density gradients. Tumour cells were found on top of the 75% Ficoll-Hypaque. TILs were found at the interface of 75% and 100% Ficoll-Hypaque. EAMNC were isolated from specimens of pleural or peritoneal effusions (Baxevanis et al, 1994*b*) and were separated from autologous tumour cells as described for TILs.

### Cell lines

Continuously growing cell lines were used as targets for assessing NK and LAK cytotoxicity. K562 (NK-sensitive) is a chronic myelogenous leukaemia cell line. Daudi (LAK-sensitive) is a Burkitt lymphoma cell line. Both cell lines were grown in culture medium RPMI-1640 (Gibco, Grand Island, NY, USA) supplemented with 10% fetal calf serum (Gibco), 2 mM L-glutamine (Sigma) and 100 μg $ml^{-1}$ gentamycin (complete medium).

### Preparation of ACD3S

PBMCs, TILs or EAMNCs ($2 \times 10^6$ cells $ml)^{-1}$ were activated in 25-$cm^2$ flasks (Costar, Cambridge, MA, USA), precoated with anti-CD3 MAb, in 5 ml of complete medium. Immobilization of anti-CD3 MAb was performed by coating the flasks with 5 μg $ml^{-1}$ MAb (anti-CD3-ε; Pharmingen, San Diego, CA, USA) as previously described (Armitage et al, 1990). After a 3- to 4-day incubation at 37°C with 5% carbon dioxide and 95% air, cultures were harvested and centrifuged. Supernatants from these cultures (ACD3S) as well as allogeneic control supernatants (ACS), which were collected from the same cultures set up in the absence of anti-CD3 MAb, were filter sterilized, aliquoted and stored at –80°C until use.

### Cytotoxicity assays

This was essentially performed as described (Baxevanis et al, 1993*b*). Briefly, effector PBMC ($2 \times 10^6$ cells $ml^{-1}$) were preincubated in 5 ml of complete medium supplemented with 25% ACD3S (or with recombinant cytokines as shown in Figure 6) in 25-ml flasks (Costar) for 3 h in carbon dioxide incubators. In blocking experiments, cytokine-specific MAbs were added to cultures, each at 10 μg $ml^{-1}$ final concentration for the entire preincubation period. Cells were then extensively washed, resuspended in fresh complete medium and placed in 100-μl aliquots into wells of 96-well V-bottom plates (Costar). Tumour targets were labelled with sodium [$^{51}$Cr]chromate (Amersham UK) and added to the effectors. The usual effector to target (E/T) ratio was 100, unless otherwise indicated. Incubation was performed for 18 h. Anti-human GM-CSF, IFN-γ, TNF-α, IL-2, IL-1β, IL-12, IL-4 MAbs were obtained from Endogen (Boston, MA, USA). Anti-human IL-5 and IL-6 MAbs were obtained from R & D systems Europe (Abingdon, UK) and anti-human IL-7 MAb was obtained from Chemicon International (Temecula, CA, USA). Anti-CD4, anti-CD8, anti-MHC class I and anti-CD18 MAbs (Chemicon), each at a final concentration of 10 μg $ml^{-1}$, were added to the effectors for 10 min before the addition of $^{51}$Cr-labelled cells. All recombinant cytokines were obtained from R & D systems, Europe.

### Quantitation of cytokines in the ACD3S

This was performed by using enzyme-linked immunosorbent assay (ELISA) kits according to the manufacturer's instructions. ELISA kits specific for IL-1β, IL-2, IL-4, IL-6 and IL-7 were obtained from R & D systems, Europe. IL-5, IL-10, IL-12, TNF-α, IFN-γ and GM-CSF were quantitated with ELISA kits from Endogen.

**Figure 4** ACD3S enhances PBMC-mediated cytotoxicity against autologous tumour cells. Autologous tumour targets were isolated from pleural malignant effusions (lung carcinoma, $n = 6$; breast carcinoma, $n = 1$) and from excised tumour specimens (breast carcinoma, $n = 6$; head and neck carcinoma, $n = 5$). ACD3S and ACS were collected from HD-derived PBMCs ($n = 9$). Mean values ± s.d. from the pooled data are given (E/T ratio = 100)

© Cancer Research Campaign 1997 

**Table 2** Quantitation of cytokine levels in ACD3S from various sources

| | ACS | ACD3S | | | |
|---|---|---|---|---|---|
| **Cytokines** | **PBMC-HD (*n* = 26)** | **PBMC-HD (*n* = 26)** | **PBMC-Ca[a] (*n* = 29)** | **TIL[a] (*n* = 22)** | **EAMNC[a] (*n* = 17)** |
| IL-1β | 55 ± 9 | 1936 ± 397[b] | 1010 ± 276[c] | 130 ± 29[d] | 110 ± 15[d] |
| IL-2 | 75 ± 23 | 5256 ± 1082[b] | 3805 ± 956[c] | 292 ± 29[d] | 237 ± 35[d] |
| IL-4 | 79 ± 26 | 997 ± 125[b] | 576 ± 139[c] | 159 ± 17[d] | 162 ± 19[d] |
| IL-5 | 45 ± 7 | 879 ± 76[b] | 327 ± 59[c] | 219 ± 122[d] | 202 ± 75[d] |
| IL-6 | 35 ± 10 | 1720 ± 537[b] | 790 ± 230[c] | 65 ± 32[d] | 90 ± 37[d] |
| IL-7 | 57 ± 23 | 1072 ± 356[b] | 570 ± 130[c] | 70 ± 13[d] | 92 ± 29[d] |
| IL-10 | 45 ± 17 | 50 ± 13 | 63 ± 25 | 270 ± 39 | 295 ± 35 |
| IL-12 | 65 ± 19 | 2176 ± 672[b] | 1180 ± 369[c] | 125 ± 29[d] | 179 ± 22[d] |
| GM-CSF | 39 ± 16 | 3577 ± 768[b] | 2590 ± 709[c] | 150 ± 20[d] | 176 ± 25[d] |
| IFN-γ | 55 ± 20 | 996 ± 190[b] | 699 ± 162[c] | 167 ± 23[d] | 196 ± 17[d] |
| TNF-α | 35 ± 19 | 1897 ± 152[b] | 1050 ± 250[c] | 63 ± 17[d] | 90 ± 23[d] |

[a]PBMCs were collected from patients with lung carcinoma (*n* = 9), ovarian carcinoma (*n* = 6), melanoma (*n* = 7) and colorectal carcinoma (*n* = 7). TILs were collected from patients with lung carcinoma (*n* = 6), melanoma (*n* = 7), breast carcinoma (*n* = 6) and renal cell carcinoma (*n* = 3). EAMNCs were collected from patients with lung carcinoma (*n* = 7), ovarian carcinoma (*n* = 7) and breast carcinoma (*n* = 3). Stimulation with immobilized anti-CD3 was performed as described in Materials and methods. Quantitation of cytokine levels was performed separately for each sample. Mean values (pg $ml^{-1}$) ± s.d. from the pooled data are given. [b] vs [c], $P < 0.05$; [c] vs [d], $P < 0.005$.

### RESULTS

PBMC collected from HD were incubated in complete medium supplemented with 25% ACD3S for the time periods indicated in Figure 1, washed three times to remove excess ACD3S and then tested as effectors against K562 (NK-sensitive) and Daudi (LAK-sensitive) tumour targets. The shortest period of incubation that produced a significant enhancement of cytotoxicity over control cultures was 3 h ($P < 0.05$). No significant additional increase of enhancement after 24 h of incubation was noticed (Figure 1). ACD3S contained no detectable amounts of anti-CD3 MAb as tested by a murine IgG-specific ELISA kit (Pharmingen; data not shown).

We and others have demonstrated that patients with advanced cancer exhibit reduced cytotoxic responses (Balch et al, 1983; Monson et al, 1987; Baxevanis et al, 1993*b*). It was therefore of interest to test the ability of ACD3S to restore such deficient responses in an effort to provide a basis for future use of ACD3S in cancer immunotherapy. As shown in Figure 2, ACD3S derived from HD-PBMCs was capable of fully restoring the NK and LAK cytotoxicity in a restricted number (*n* = 7) of cancer patients at all E/T ratios. These findings were also extended to a higher number of patients. As presented in Table 1, ACD3S greatly increased the killing of both K562 and Daudi tumour targets by effector PBMCs from 90 patients with different types of cancer. The percentages of enhancement ranged between 39% and 80% for NK cytotoxicity and between 54% and 185% for LAK cytotoxicity. Preincubation of PBMC in plain medium for 3 h resulted in equal levels of cytotoxicity (not shown) to those obtained upon preincubation with control supernatants (ACS) collected from the same PBMC cultures in the absence of anti-CD3 (Table 1).

It has been reported that mononuclear cells freshly isolated from the tumour environment exhibit decreased immunological parameters in terms of proliferation and cytotoxicity (Miescher et al, 1988; Alexander et al, 1993). Thus, we examined whether this unresponsiveness in TILs and EAMNCs would also result in production of inactive supernatants upon stimulation with immobilized anti-CD3 MAbs. Indeed, ACD3S derived from TILs (melanoma, breast carcinoma, head and neck carcinoma, *n* = 7) and EAMNCs (lung carcinoma, ovarian carcinoma, *n* = 6) did not significantly enhance the killing of Daudi tumour targets upon preincubation for 3 h with either HD- or patient-derived PBMCs (Figure 3). Even prolonged preincubation (24 h) or higher doses (complete medium supplemented with 50% of such supernatants) did not improve the cytotoxicity levels (data not shown). In contrast, stimulation of patients' PBMCs (*n* = 9) with immobilized anti-CD3 MAb resulted in ACD3S that significantly enhanced the killing of Daudi targets by both HD- and patient-derived PBMCs ($P < 0.01$; Figure 3). However, this enhancement was significantly lower than that induced by ACD3S derived from HD-PBMCs ($P < 0.05$; Figure 3).

As cellular adoptive immunotherapy of cancer is based on the eradication of autologous tumour cells in vivo by ex vivo-activated cytotoxic lymphocytes, we were interested in determining whether ACD3S were capable of enhancing in vitro the killing of tumour cells by autologous PBMCs. The data in Figure 4 clearly show that ACD3S from HD-derived PBMCs induced a two- to three-fold increase of autologous tumour-cell killing in all 18 cases tested. In these experiments (and those of Figures 5–7), tumour cells isolated from surgically excised tumour specimens or malignant (pleural, peritoneal) effusions were kept frozen in liquid nitrogen until blood was withdrawn from the same patients for isolation of autologous PBMCs.

There are numerous reports demonstrating the involvement of various cytokines alone or in synergy with IL-2 in cytotoxic responses against tumour cells (Owen-Schaub et al, 1988; Aoki et al, 1989; Naume and Espevik, 1991; Fujiwara and Grimm, 1992; Baxevanis et al, 1995). On the other hand, the data presented herein show that there are significant differences between ACD3S from HD-derived PBMCs and cancer patient-derived PBMCs, TILs or EAMNCs, with respect to their capacity to induce enhanced cytotoxicity (Figure 3). Thus, it was of interest to correlate the potency of such supernatants to enhance killing of tumour cells with the levels of certain cytokines that are involved in this type of response. Cytokine analyses were performed in a large number of samples for every group to allow statistical comparison (Table 2). High levels of various cytokines could be detected in all ACD3S from healthy donors. Increased levels of the same cytokines were also detected in the ACD3S from cancer

 © Cancer Research Campaign 1997

**Figure 5** Cytokine-specific MAbs reduce the ACD3S-induced enhancement of cytotoxic responses. MAbs were present throughout the preincubation period of PBMCs with ACD3S. Thereafter, PBMCs were washed three times to remove residual of supernatant and MAb and used as effectors in the cytotoxic assays. ACD3S were the same as those of Table 2. Autologous tumour cells were isolated from malignant peritoneal (ovarian carcinoma, $n = 5$) and pleural (breast carcinoma, $n = 2$; lung carcinoma, $n = 3$) effusions. Pooled data ± sd are shown. PBMCs from the same patients were used as effectors. Inhibition was statistically significant ($P < 0.05$), except with anti-IL-4 and anti-IL-5 MAbs

**Figure 6** Superior immunopotentiating effect of ACD3S to simple incubation of PBMCs with each recombinant cytokine alone. Patients' PBMCs (lung carcinoma, $n = 2$; breast carcinoma, $n = 2$; ovarian carcinoma, $n = 2$) were incubated for 3 h with ACD3S or each of the recombinant cytokines and then tested for cytotoxicity against Daudi or autologous tumour cells. ACD3S was collected from HD-derived PBMCs ($n = 5$). First, second, third and fourth doses for each of the recombinant cytokines used were, respectively: 1000, 100, 10 and 1 IU ml$^{-1}$ for IL-2; 100, 10, 1 and 0.1 ng ml$^{-1}$ for IL-1, IL-6 and IL-12; 200, 20, 2 and 0.2 ng ml$^{-1}$ for GM-CSF, IFN-γ and TNF-α (final concentrations)

patient-derived PBMCs which, however, were significantly lower than those from healthy donors (Table 2). In agreement with the data of Figure 3, there was a tremendous decrease in the amounts of these cytokines in ACD3S from TILs and EAMNCs. Interestingly, fivefold higher levels of IL-10 could be measured in ACD3S from TILs and EAMNCs, which may be associated with their inability to stimulate anti-tumour cytotoxicity in PBMCs.

To directly correlate the presence of the above cytokines with the ability of ACD3S to enhance the PBMC-mediated cytotoxicity, we performed blocking experiments by adding cytokine-specific MAbs along with the ACD3S for the duration of the 3-h preincubation. MAb against IL-1β, IL-2, IL-6, IL-7, IL-12, GM-CSF, IFN-γ and TNF-α significantly reduced the ACD3S-induced enhanced cytotoxicity against Daudi (reduction range 35–50%) and autologous tumour targets (reduction range 36–56%) (Figure 5). In the same experiments MAbs against IL-4 and IL-5 remained without any significant effect, suggesting that both cytokines did not contribute to the observed enhancement of cytotoxicity. Even at higher doses (20 or 40 μg ml$^{-1}$), anti-IL-4 and anti-IL-5 MAbs did not effectively inhibit the ACD3S-mediated augmented effect (data not shown). These results confirm that a synergy of certain cytokines, present in excess within the ACD3S, is responsible for the observed enhancement of the anti-tumour cytotoxic responses. The superior immunostimulatory effect of the ACD3S to simple incubation with each of these cytokines alone was documented in

© Cancer Research Campaign 1997

**Figure 7** CD18-dependent lysis of tumour targets by ACD3S-activated PBMCs. MAbs were added to the effectors 10 min before the addition of the $^{51}$Cr-labelled tumour targets. Patients and ACD3S were the same as in Figure 5. Inhibition with anti-CD18 MAb was statistically significant ($P < 0.01$)

the next series of experiments. As shown in Figure 6, none of the cytokines alone could enhance killing of Daudi or autologous tumour targets by patients' PBMCs. Only IL-2 at a high dose (1000 IU $ml^{-1}$) could promote cytotoxicity to levels that, however, were significanty lower than those achieved with ACD3S (20% vs 33% killing against Daudi and 17% vs 32% killing against autologous tumour targets; in both cases, $P < 0.05$).

Cytolytic activity depends on the binding of the effector lymphocytes to the tumour target cells. In this process, accessory, MHC and adhesion molecules have been reported to be involved (Baxevanis and Papamichail, 1994; Gamero et al, 1995). To test which molecules are involved in the recognition of tumour targets by the ACD3S-activated effectors, MAbs against CD4, CD8, MHC class I and CD18 molecules were added to the lytic assay to examine their ability to inhibit cytotoxic function. Experiments from six patients demonstrate that the ACD3S-induced cytotoxic responses against both Daudi and autologous tumour targets were not MHC-restricted, as none of the anti-CD4, anti-CD8 or anti-MHC class I MAbs showed an inhibitory effect. In contrast, anti-CD 18 MAb markedly inhibited (average inhibition 51%) lysis of Daudi and autologous tumour cells (Figure 7). These data suggest that lysis of tumour targets by ACD3S-activated PBMCs is mediated by non-MHC-restricted cells whereby the CD18 adhesion molecule plays a critical role.

### DISCUSSION

The data presented herein describe the ability of supernatants from cultures of healthy donor-derived PBMCs with immobilized anti-CD3 MAb (ACD3S) to induce, upon 3-h preincubation, tumour-reactive cytotoxic lymphocytes among PBMCs from cancer patients with advanced disease. Cytotoxicity was directed against both allogeneic tumours or tumour cell lines and autologous tumour cells. A number of cytokines, such as IL-1β, IL-2, IL-6, IL-7, IL-12, GM-CSF, IFN-γ and TNF-α present at high titres within the ACD3S, were responsible for the induction of cytotoxicity. This was shown by two different means: (1) cytokine-specific antibodies added to the effector PBMCs in the presence of ACD3S diminished, to a great extent, the enhancement of cytotoxicity; and (2) ACD3S derived from TILs and EAMNCs contained drastically lower (six- to 30-fold) quantities of the above mentioned cytokines and accordingly produced little if any effect. The fact that IL-4 and IL-5, although detected at increased levels within the ACD3S, did not significantly influence the observed enhancement of cytotoxicity may simply be as a result of the culture system used. We have shown that 3 h of incubation yields a prominent enhancement of cytotoxicity that does not significantly differ from that obtained upon 24-h preincubation (Figure 1). Therefore, we believe that incubation of PBMC with ACD3S over 24 h, in an effort to detect any IL-4- and IL-5-mediated enhancing effects, would not additionally serve the aim of the present study.

ACD3S from TILs and EAMNCs contained increased levels of IL-10. This cytokine can suppress the secretion of other cytokines, such as IFN-γ, IL-1β, IL-6 and TNF-α (Fiorentino et al, 1991; DeWaal-Malefyt et al, 1993; Taga et al, 1993) and therefore may be responsible for the low titre of cytokines present within the TIL- and EAMNC-derived ACD3S. Neutralizing the biological activity of IL-10 by anti-IL-10 MAb during ACD3S production may clarify its role in this sytem. On the other hand, it is known that freshly isolated TILs and EAMNCs are functionally impaired, with abnormal in vitro proliferation to polyclonal activators (Miescher et al, 1987, 1988; Alexander et al, 1993). This lack of responsiveness could also account for the low cytokine levels within such supernatants. In contrast to TILs and EAMNCs, PBMCs from cancer patients were able to produce biologically active ACD3S with elevated titres of the relevant cytokines. However, the enhancing effect induced by such supernatants was significantly weaker than that of HD-PBMCs. This was not because of the levels of IL-10 as these were almost equally low in ACD3S from both groups, but rather to the levels of the crucial cytokines (i.e. IL-1β, -2, -6, -7, -12 and GM-CSF, IFN-γ and TNF-α), which were significantly lower (1.4- to 2.2-fold) in ACD3S from patients' PBMCs. In accordance with this, preliminary data from our laboratory suggest that such supernatants can be as effective as those from healthy donors when their content in culture is increased. Cellular immunity in cancer patients with advanced disease has been reported to be impaired in several aspects, including T-cell activation and proliferation, to

© Cancer Research Campaign 1997

a variety of stimuli (Monson et al, 1987; Anastasopoulos et al, 1992; Kosmidis et al, 1992; Baxevanis et al, 1993*a,c*; Such deficiencies may contribute to the production of less active supernatants by patients' PBMCs.

There are numerous reports demonstrating the capacity of anti-CD3 MAb to induce cytotoxic responses in a variety of systems. Suthanthiran et al (1984) were the first to show that pretreatment of human alloreactive memory cells, generated in primary mixed lymphocyte cultures, with anti-CD3 resulted in the induction of specific secondary cytolytic activity and natural killer cell-like activity. Murine TILs sequentially activated with solid-phase anti-CD3 produced increased levels of IFN-γ and GM-CSF in vitro and were capable of eradicating pulmonary metastases induced by the MCA-105 sarcoma in vivo (Geodegebuure et al, 1994). In another study (Katsanis et al, 1994), short-term ex vivo activation of splenocytes with anti-CD3 plus IL-2 and infusion post-bone marrow transplantation into mice resulted in in vivo expansion of effector cells with potent anti-lymphoma activity. In vivo administration of anti-CD3 MAb plus IL-2, induced intrahepatic expression of mRNA-encoding perforin, cytotoxic T-cell-specific serine esterase and TNF-α that resulted in a significantly smaller number of hepatic metastases and significantly longer survival time of tumour-bearing mice (Nakajama et al, 1994). In humans, immobilized anti-CD3, along with IL-2, has successfully been used for large-scale expansion of PBMCs with non-MHC-restricted cytotoxicity for adoptive cellular immunotherapy after bone marrow transplantation (Uberti et al, 1994). Immobilized anti-CD3, along with IL-2, has been demonstrated to induce high cytotoxicity against autologous tumour cells in cytotoxic T-cells generated during an autologous mixed lymphocyte tumour-cell culture (Tani et al, 1995). Anti-CD3 has also been shown to stimulate LAK cells to lyse acute myeloid leukaemia cells (Kaneko et al, 1994) and to induce in vitro expansion and activation of mucin-reactive T-helper lymphocytes from patients with colorectal cancer (Kim et al, 1995). Finally, in a recent clinical study (Goedegebuure et al, 1995), TILs activated with anti-CD3 MAb for 48 h and expanded in low-dose IL-2 in vitro produced high levels of IL-6, GM-CSF, TNF-α and IL-4 with improved clinical results in patients with melanoma and renal cell carcinoma.

Our results yield additional information regarding the ability of anti-CD3 MAb to induce cytotoxic responses. We consistently measured high levels of IL-1β, -2, -6, -7, -12 and GM-CSF, TNF-α and IFN-γ in the ACD3S from all 55 donor PBMCs tested (26 healthy donor and 29 carcinoma PBMC). All these cytokines have previously been shown to be potent inducers of anti-tumour cytotoxicity by activating both effectors with specificity against autologous tumours and effectors that lyse a broad spectrum of allogeneic tumours (Mule et al, 1987; Fujiwara and Grimm, 1992; Porgador et al, 1993; Nostala et al, 1994; Baxevanis et al, 1995). To our knowledge, this is the first report that demonstrates such a synergy between endogenously produced cytokines in the induction of tumour-reactive lymphocytes within only 3 h of incubation. The term 'synergy' is applied because (1) none of the cytokine-specific MAbs used was able to completely block the response and (2) none of these cytokines alone, with the exception of IL-2 at high doses, was able upon 3-h incubation to increase the patients' PBMCs cytotoxic capacity. The lytic activity induced by ACD3S in patients' PBMCs is non-MHC-restricted and at least partly CD18 dependent. The non-MHC-restricted lysis was demonstrated by two means: (1) by the fact that anti-CD4, anti-CD8 and anti-MHC class I MAbs failed to inhibit lysis against autologous tumour cells and Daudi cells; (2) ACD3S-activated effectors were able to lyse K562 cells, which are MHC class I negative, as well as Daudi cells and primary allogeneic tumour cells, which express different MHC class I molecules. The initiation of the cytolytic process requires contact of the tumour cells with the effector cells, and an essential component is the interaction of ICAM-1 on the tumour targets with CD18 on the effectors (Blauchard et al, 1990). Our data demonstrate that this molecule also participates in the cytotoxic process induced by ACD3S against autologous and allogeneic tumour cells, as well as against tumour cell lines. As the lytic activity enhanced by ACD3S is non-MHC restricted, CD18 must be expressed on activated $CD56^+$ cells, which mediate this type of cytotoxicity (Baxevanis and Papamichail, 1994).

In conclusion, we demonstrate herein that cytokine-rich supernatants produced by anti-CD3-activated HD-PBMCs during a 3-h incubation can induce cancer patients' lymphocytes to lyse a variety of tumour targets, including autologous tumour cells. These data suggest the therapeutic use of the application of such supernatants for a short-term activation of patients' PBMCs collected with leucapheresis. Mixtures of recombinant cytokines (such as those detected in the ACD3S) may also be used in this respect. We believe that this approach offers several advantages over that introduced by Osband et al (1990), which is based on the use of supernatants derived from activated autologous (cancer patient derived) PBMCs. First, ACD3S are derived from PBMCs of (allogeneic) healthy donors which, as shown herein, are superior to those collected from cancer patients in terms of inducing cytotoxicity against tumour targets, including patients' tumours. Second, ACD3S can be collected easily from healthy donor-derived PBMCs, pooled at high quantities and stored until use. In this way, sufficient material (ACD3S) will be available at any time for immunotherapeutic trials. In addition, this approach offers a less costly method of cancer immunotherapy using ex vivo-activated effector lymphocytes with non-MHC-restricted cytotoxicity. This method could make cellular adoptive immunotherapy more accessible to cancer patients and reduce the dependency of therapy on the availability of a large-scale culture facility as well as the reported side-effects that are associated with this type of therapy.

### ACKNOWLEDGEMENT

We wish to thank Miss Joanna Doukoumopoulou for her excellent secretarial assistance.

### REFERENCES

Alexander JP, Kudoh S, Meslop KA, Hamilton TA, Edinger MG, Tubbs RR, Sica D, Tuason L, Klein E and Bukowski RM (1993) T-cells infiltrating renal-cell carcinoma display a poor proliferation response even they can produce interleukin 2 and express interleukin 2 receptors. *Cancer Res* **53**: 1380–1387

Anastasopoulos E, Reclos GJ, Baxevanis CN, Gritzapis AD, Tsilivakos V, Panagiotopoulos N, Fotiou S, Missitzis J, Karydas E and Papamichail M (1992) Monocyte disorders associated with T cell defects in patients with solid tumors. *Anticancer Res* **12**: 489–494

Aoki T, Kikuchi H, Miyatake SI, Oda Y, Iwasaki K, Yamasaki T, Kinashi T and Honjo T (1989) Interleukin-5 enhances interleukin-2-mediated lymphokine-activated killer activity. *J Exp Med* **170**: 583–592

Armitage RJ, Namen AE, Sassenfeld HM and Grabstein KH (1990) Regulation of human T cell proliferation by IL-7. *J Immunol* **144**: 938–941

Balch CM, Tilden AB, Dougherty PA, Cloud G and Abu T (1983) Depressed levels of granular lymphocytes with natural killer (NK) cell function in 247 cancer patients. *Ann Surg* **198**: 192–199

© Cancer Research Campaign 1997

Baxevanis CN and Papamichail M (1994) Characterization of the anti-tumor immune response in human cancers and strategies for immunotherapy. *Crit Rev Oncol Hematol* **16**: 157–179

Baxevanis CN, Reclos GJ, Gritzapis AD, Dedoussis GVZ, Missitzis I and Papamichail M (1993*a*) Elevated prostaglandin E2 production by monocytes is responsible for the depressed levels of natural killer and lymphocyte-activated killer cell function in patients with breast cancer. *Cancer* **72**: 491–501

Baxevanis CN, Reclos GJ and Papamichail M (1993*b*) Prothymosin-α restores depressed allogeneic cell-mediated lympholysis and natural-killer-cell activity in patients with cancer. *Int J Cancer* **53**: 264–268

Baxevanis CN, Reclos GJ, Gritzapis AD, Dedoussis GVZ, Arsenis P, Katsiyiannis A, Mitsis PG, Tsavaris N and Papamichail M (1993*c*) Comparison of immune parameters in patients with one or two primary malignant neoplasms. *Nat Immun* **12**: 41–49

Baxevanis CN, Dedoussis GVZ, Papadopoulos NG, Missitzis I, Stathopoulos GP and Papamichail M (1994*a*) Tumor specific cytolysis by tumor infiltrating lymphocytes in breast cancer. *Cancer* **74**: 1275–1282

Baxevanis CN, Dedoussis GVZ, Gritzapis AD, Stathopoulos GP and Papamichail M (1994*b*). Interleukin-1β synergizes with interleukin 2 in the outgrowth of autologous tumor-reactive CD8+ effectors. *Br J Cancer* **70**: 625–630

Baxevanis CN, Dedoussis GVZ, Papadopoulos NG, Missitzis I, Beroukas C, Stathopoulos GP and Papamichail M (1995) Enhanced human lymphokine-activated killer cell function after brief exposure to granulocyte–macrophage-colony stimulating factor. *Cancer* **76**: 1253–1260

Blanchard DK, Hall RE and Djeu JY (1990) Role of CD18 in lymphokine activated killer (LAK) cell-mediated lysis of human monocytes: comparison with other LAK targets. *Int J Cancer* **45**: 312–329

Curti BD, Longo DL, Ochoa AC, Conlon KC, Smith JW, Alyord G, Creekmores SP, Fenton RG, Gause BL and Holmlund J (1993) Treatment of cancer patients with ex vivo anti-CD3-activated killer cells and interleukin-2. *J Clin Oncol* **11**: 652–660

Dranoff G, Jaffee E, Lazenby A, Golenmbek P, Levitsky H and Brose K (1993) Vaccination with irradiated tumor-cells engineered to secrete murine granulocyte–monocyte colony-stimulating factor stimulates potent, specific, and long-lasting anti-tumor immunity. *Proc Natl Acad Sci USA* **90**: 3539–3543

Ettinghausen SE and Rosenberg SA (1995) Immunotherapy and gene therapy of cancer. *Adv Surg* **28**: 223–254

Fiorentino DF, Zlotnik A, Mossmann TR, Howard M and O'Garra A (1991) IL-10 inhibits cytokine production by activated macrophages. *J Immunol* **147**: 3815–3822

Fujiwara T and Grimm EA (1992) Regulation of lymphokine-activated killer cell induction by human recombinant IL-1 receptor antagonist. *J Immunol* **148**: 2941–2946

Gamero AM, Ussery D, Reintgen DS, Puleo CA and Djeu JY (1995) Interleukin 15 induction of lymphokine-activated killer cell function against autologous tumor cells in melanoma patient lymphocytes by a CD18-dependent, perforin-related mechanism. *Cancer Res* **55**: 4988–4994

Gately MK, Gubler U, Brunda MJ, Nadeau RR, Anderson TD, Lipman JM and Sarmiento U (1994) Interleukin-12: a cytokine with therapeutic potential in oncology and infectious diseases. *Ther Immunol* **1**: 187–196

Geodegebuure PS, Zuber M, Leonard-Vidal DL, Burger UL, Cusak JC and Chang MP (1994) Reactivation of murine tumor-infiltrating lymphocytes with solid-phase anti-CD3 antibody: in vitro cytokine production is associated with in vivo efficacy. *Surg Oncol* **3**: 79–89

Goedegebuure PS, Douville LM, Li H, Richmond GC, Shoof DD, Scavone M and Eberlein TJ (1995) Adoptive immunotherapy with tumor-infiltrating lymphocytes and interleukin-2 in patients with metastatic malignant melanoma and renal cell carcinoma: a pilot study. *J Clin Oncol* **13**: 1939–1949

Kaneko T, Fusauch Y, Kakni Y, Okumura K, Mizoguchi H and Oshimi K (1994) Cytotoxicity of cytokine-induced killer cells coated with bispecific antibody against acute myeloid leukemia cells. *Leuk Lymphoma* **14**: 219–229

Katsanis E, Xu Z, Anderson PM, Dancisak BB, Bausero MA, Weisdorf DJ, Blazar BR and Ochoa AC (1994) Short-term ex vivo activation of splenocytes with anti-CD3 plus IL-2 and infusion post-BMT into mice results in in vivo expansion of effector cells with potent anti-lymphoma activity. *Bone Marrow Transplant* **14**: 563–572

Kim JA, Martin EW, Morgan CJ, Aldrich W and Triozzi PL (1995) Expansion of mucin-reactive T-helper lymphocytes from patients with colorectal cancer. *Cancer Biother* **10**: 115–123

Kosmidis PA, Baxevanis CN, Tsavaris N, Papanastasiou M, Anastasopoulos E, Bacoyiannis C, Mylonakis N, Karvounis N, Bafaloukos SD, Karabellis A and Papamichail M (1992) The prognostic significance of immune changes in patients with renal cell carcinoma treated with interferon alpha-2b. *J Clin Oncol* **7**: 1153–1157

Mehrotra PT, Grant AJ and Siegel JP (1995) Synergistic effects of IL-7 and IL-12 on human T cell activation. *J Immunol* **154**: 5093–5102

Miescher S, Whiteside TL, Moretta L and von Fliedner V (1987) Clonal and frequency analysis of tumor-infiltrating T lymphocytes from human solid tumors. *J Immunol* **138**: 4004–4011

Miescher S, Stoeck M, Qiao L, Barras C, Barrelet L and von Fliedner V (1988) Proliferative and cytolytic potentials of purified human tumor-infiltrating T-lymphocytes. Impaired response to mitogen-driven stimulation despite T-cell receptor expression. *Int J Cancer* **42**: 659–666

Monson JRT, Ramsden CW, Giles GR, Brennan TG and Guillon PJ (1987) Lymphokine-activated killer (LAK) cells in patients with gastrointestinal cancer. *Gut* **28**: 420–425

Mule JJ, Smith GA and Rosenberg SA (1987) Interleukin-4 can mediate the induction of lymphokine-activated killer cell activity directed against fresh tumor cells. *J Exp Med* **166**: 792–803

Nakajama F, Khanna A, Xu G, Lagman M, Haschemeyer R, Mouradian J, Wang JC, Stenzel KH, Rubin AL and Suthanthiran M (1994) Immunotherapy with anti-CD3 monoclonal antibodies and recombinant interleukin 2: Stimulation of molecular programs of cytotoxic killer cells and induction of tumor regression. *Proc Natl Acad Sci USA* **91**: 7889–7893

Nostala CG, Edington HD, McKinney TG, Tahera H, Nalesnik MA, Brunda MJ, Gately MK, Wolf SF, Schreiber RD and Storkus WJ (1994) Recombinant IL-12 administration induces tumor regression in association with IFN-γ production. *J Immunol* **153**: 1697–1706

Naume B and Espevik T (1991) Effects of IL-7 and IL-12 on highly enriched CD56+ natural killer cells: a comparative study. *J Immunol* **147**: 2208–2213

Osband ME, Lavin PT, Babayan RK, Graham S, Lamm DL, Parker B, Sawczuki Ross S and Krane RJ (1990) Effect of autolymphocyte therapy on survival and quality like in patients with metastatic renal-cell carcinoma. *Lancet* **335**: 994–998

Owen-Schaub LB, Gutterman JV and Grimm EA (1988) Synergy of tumor necrosis factor and interleukin 2 in the activation of human cytotoxic lymphocytes: effect of tumor necrosis factor alpha and interleukin-2 in the generation of human-activated killer cell cytotoxicity. *Cancer Res* **48**: 788–794

Papamichail M and Baxevanis CN (1992) Gamma-interferon enhances the cytotoxic activity of interleukin-2-induced LAK cells, TIL, and effusion-associated lymphocytes. *J Chemother* **4**: 387–392

Porgador A, Bannerji R, Watanabe Y, Feldman Y, Gilboa E and Eisenbach L (1993) Antimetastatic vaccination of tumor-bearing mice with two types of IFN-γ gene-inserted tumor cells. *J Immunol* **150**: 1458–1470

Sone S, Utsugi T, Mii A and Ogura T (1988) Differential effects of recombinant interferon alpha, beta, and gamma on the induction of human lymphokine (IL-2)-activated killer activity. *J Natl Cancer Inst* **80**: 425–430

Suthantiran M, Williams PS, Solomon SD, Rubin AL and Stenzel KH (1984) Induction of cytolytic activity by anti-T3 monoclonal antibody. Activation of alloimmune memory cells and natural killer cells from normal and immunodeficient individuals. *J Clin Invest* **74**: 2263–2271

Taga K, Mostowski H and Tosato G (1993) Human interleukin 10 can directly inhibit T-cell growth. *Blood* **81**: 2964–2971

Tani M, Tanimura H, Yamane H, Mizobata S, Iwahashi M, Tsunoda T, Noguchi K, Tamai M, Hotta T and Teresawa H (1995) Generation of CD4+ cytotoxic T lymphocytes stimulated by immobilized anti-CD3 monoclonal antibody and interleukin-2 in cancer patients. *Int J Cancer* **60**: 802–807

Uberti JP, Joshi I, Ueda M, Martilotti F, Sensenbrenner LL and Lum LG (1994) Preclinical studies using immobilized OKT3 to activate human T cells for adoptive immunotherapy: optimal conditions for the proliferation and induction of non MHC-restricted cytotoxicity. *Clin Immunol Immunopathol* **70**: 234–240

Ubhi SS, Horsburgh T, Veitch PS and Bell PRF (1991) Anti-CD3 enhancement of cellular cytotoxicity in cancer patients treated with interleukin-2. *Anticancer Res* **11**: 931–936

Ullman KS, Northrop JP, Verweji CL and Crabtree GR (1990) Transmission of signals from the T lymphocyte antigen receptor to the genes responsible for cell proliferation and immune function: the missing link. *Annu Rev Immunol* **8**: 421–452

de Waal-Malefyt R, Yssel H and De Vries JE (1993) Direct effects of IL-10 subsets of human CD4+ T cell clones and resting T cell. Specific inhibition of IL-2 production and proliferation. *J Immunol* **150**: 4754–4765

Yoshizawa H, Chang AE and Shu S (1992) Cellular interactions in effector cell generation and tumor regression mediated by anti-CD3/interleukin-2-activated tumor-activated tumor-draining lymph node cells. *Cancer Res* **52**: 1129–1136

© Cancer Research Campaign 1997